# A Kalman Filter-Based Short Baseline RTK Algorithm for Single-Frequency Combination of GPS and BDS

**DOI:** 10.3390/s140815415

**Published:** 2014-08-20

**Authors:** Sihao Zhao, Xiaowei Cui, Feng Guan, Mingquan Lu

**Affiliations:** Department of Electronic Engineering, Tsinghua University, No.1 Qinghuayuan, Haidian District, Beijing 100084, China; E-Mails: cxw2005@tsinghua.edu.cn (X.C.); guanfeng@163.com (F.G.); lumq@tsinghua.edu.cn (M.L.)

**Keywords:** real time kinematic (RTK) algorithm, Global Positioning System (GPS), BeiDou navigation satellite system (BDS), Kalman filter, ambiguity resolution

## Abstract

The emerging Global Navigation Satellite Systems (GNSS) including the BeiDou Navigation Satellite System (BDS) offer more visible satellites for positioning users. To employ those new satellites in a real-time kinematic (RTK) algorithm to enhance positioning precision and availability, a data processing model for the dual constellation of GPS and BDS is proposed and analyzed. A Kalman filter-based algorithm is developed to estimate the float ambiguities for short baseline scenarios. The entire work process of the high-precision algorithm based on the proposed model is deeply investigated in detail. The model is validated with real GPS and BDS data recorded from one zero and two short baseline experiments. Results show that the proposed algorithm can generate fixed baseline output with the same precision level as that of either a single GPS or BDS RTK algorithm. The significantly improved fixed rate and time to first fix of the proposed method demonstrates a better availability and effectiveness on processing multi-GNSSs.

## 1. Introduction

Global Navigation Satellite Systems (GNSSs) have been extensively adopted to provide accurate and continuous positioning, navigation and timing (PNT) services on a global scale. Among GNSSs, China's BeiDou Navigation Satellite System (BDS) has achieved its regional coverage and it has been providing PNT services over China and its surrounding areas since the end of 2012 [1,2]. The regional BDS constellation is comprised of five geo-stationary Earth orbit (GEO) satellites, five inclined geosynchronous orbit (IGSO) satellites and four medium Earth orbit (MEO) satellites and will evolve into a constellation with 5 GEOs + 3 IGSOs + 27 MEOs by 2020 to realize its global service [3,4].

A GNSS receiver senses the carrier's position information, whose precision can be significantly improved with a multitude of carrier phase based techniques among which the real time kinematic (RTK) algorithm is known for its real-time and high-precision features. Compared with single point positioning using only pseudorange measurements, RTK utilizes the signal carrier phase information which is more precise than the pseudorandom code measurements and has the potential to diminish the position error to centimeter level [5]. Another feature is its use of real-time broadcast satellite navigation messages other than post-processed precise ephemeris products frequently adopted in non-real-time applications including relative static positioning that requires higher precision and precise point positioning (PPP). However, RTK requires a higher quality and a larger number of received navigation signals than pseudorange-based positioning methods to guarantee its high-precision and continuous performance which limits its availability and reliability. With the combination of GPS and BDS, the number of satellites available for users is increased, which has a potential to improve the positioning precision and availability for RTK. Motivated by the fast development of BDS, recent research works have begun to study the feasibility and methods to exploit the ability of BDS for real time high-precision positioning. The contribution in [6] utilized simulated single-epoch measurements to evaluate the RTK performance on BDS multi-frequency signals. The raw measurement characteristics are analyzed and the single point and short baseline relative positioning results were demonstrated in [7]. Another study in [8] focused on the measurement quality of BDS GEO and IGSO satellites and compared the single point and relative positioning performance between BDS and GPS. The research of [9] illustrated the RTK performance improvement of a combination of BDS and GPS over a single system with a single-epoch ambiguity resolution method. Work in [10] analyzed the single-baseline observation model and assessed the relative positioning performance of GPS+BDS with also a single-epoch ambiguity resolution.

In this work, we first analyze the measurement model of double-differenced (DD) carrier phases and pseudoranges based on which a Kalman filtering model that utilizes previous and current measurement information to estimate the carrier phase float ambiguities is proposed. This model can process carrier phase and pseudorange measurements from a sole constellation of BDS or GPS. We adapt the original model to the dual-constellation case and use measurements from both systems as input to the Kalman filter to estimate all float ambiguities and their variances, and then we resolve the integer ambiguities of the two constellations simultaneously. Due to the larger number of visible satellites than either single constellation, this combined method has the potential to increase the availability of RTK. We also propose an implementation scheme with details including filter parameter setting, satellite rising/setting handling, baseline resolution, *etc.*, which is rarely found in previous literature, and realize it with Matlab code to process the double-differenced measurements from the two systems with different signal frequencies and time frames. Tests using field-collected single-frequency measurement data of GPS and BDS generated by our self-developed receivers from a zero baseline and two short baseline experiments are conducted to validate the proposed model. Results show that high-precision baseline solutions with the same precision level as a sole GPS or BDS RTK method can be obtained by processing the dual-constellation measurements with the proposed model, the percentage of integer ambiguities and fixed baseline in all epochs using double-constellation is higher than using either single constellation, and a shorter convergence time to first fix can be obtained as well. This proposed model with implementation details can help the reader to realize his/her own application and also can be extended to multi-constellation and multi-frequency scenarios and can be a guidance for future applications which is the main contribution of this research work.

In the following parts of this paper, we first introduce the GNSS carrier phase and pseudorange measurement model as the foundation of the analysis and derivations in the remaining sections. Next, a Kalman filter-based float ambiguity estimation algorithm is proposed and improved to process the combined GPS and BDS measurements. The architecture and work process of the model are developed and presented in detail. In the experiment part, a zero baseline and two short baseline field tests to validate the proposed model are conducted from which both carrier phase and pseudorange raw measurements of GPS L1 and BDS B1 frequencies are collected. These data are processed by the proposed model to produce baseline and ambiguity solutions. The results from both single GPS and BDS and their combination are compared and analyzed. Finally, a conclusion is drawn and suggestions for future work are presented.

## 2. Carrier Phase and Pseudorange Measurement Model

Under a single baseline condition, the GNSS receivers at both ends are named “base” and “rover” (subscripts “b” and “r” are used in equations hereinafter) respectively in accordance with literature. The carrier phase and pseudorange measurements from a certain satellite *j* of an arbitrary constellation observed by the two receivers at a certain epoch can be written as

(1) $$\rho_{j,\text{r}}=\lambda^{-1}\left(r_{j,\text{r}}+I_{j,\text{r}}+T_{j,\text{r}}\right)+f\left(\delta t_{\text{r}}-\delta t_{j}\right)+{ɛ}_{j,\text{r}}$$

(2) $$\rho_{j,\text{b}}=\lambda^{-1}\left(r_{j,\text{b}}+I_{j,\text{b}}+T_{j,\text{b}}\right)+f\left(\delta t_{\text{b}}-\delta t_{j}\right)+{ɛ}_{j,\text{b}}$$

(3) $$\phi_{j,\text{r}}=\lambda^{-1}\left(r_{j,\text{r}}-I_{j,\text{r}}+T_{j,\text{r}}\right)+f\left(\delta t_{\text{r}}-\delta t_{j}\right)+N_{j,\text{r}}+\eta_{j,\text{r}}$$

(4) $$\phi_{j,\text{b}}=\lambda^{-1}\left(r_{j,\text{b}}-I_{j,\text{b}}+T_{j,\text{b}}\right)+f\left(\delta t_{\text{b}}-\delta t_{j}\right)+N_{j,\text{b}}+\eta_{j,\text{b}}$$

where *ρ* and ϕ are pseudorange and carrier phase measurements (unit: carrier cycle), respectively, *λ* is the carrier wavelength (unit: m), *r* represents the true geographical distance between the satellite and the receiver (unit: m), *T* is the tropospheric delay (unit: m), *I* is the ionospheric delay (unit: m), *f* is the carrier frequency (unit: Hz), *δt*_r_ is the receiver clock error (unit: s), *δt*_j_ is the satellite clock error (unit: s), *N* is the integer ambiguity, *ε* and *η* are measurement errors of pseudorange and carrier phase respectively and their variances can be modeled as a simplified function of the elevation angle based on [11] as given by Equation (5):

(5) $$\sigma^{2}=a\left(b+b/\sin^{2}\theta \right)/\lambda^{2}$$

where *θ* is the elevation angle of the satellite, *a* and *b* can be set empirically, for example, we select *a* = 1 and *b* = 9e − 6 for carrier phase in the following experiment part.

The single-differenced (SD) measurement model can be obtained by subtracting the base receiver's from the rover receiver's measurements, *i.e.*, Equation (1) − Equation (2) and Equation (3) − Equation (4):

(6) $$\rho_{j,\text{rb}}=\lambda^{-1}\left(r_{j,\text{rb}}+I_{j,\text{rb}}+T_{j,\text{rb}}\right)+f\delta t_{\text{rb}}+{ɛ}_{j,\text{rb}}$$

(7) $$\phi_{j,\text{rb}}=\lambda^{-1}\left(r_{j,\text{rb}}-I_{j,\text{rb}}+T_{j,\text{rb}}\right)+f\delta t_{\text{rb}}+N_{j,\text{rb}}+\eta_{j,\text{rb}}$$

where the subscript “rb” represents the difference between the corresponding terms of rover and base. It can be seen that *δt*_j_ as a common error is eliminated by this differencing.

Next, we choose the *i*th satellite with the highest elevation angle as reference, and the double differences of carrier phases and pseudoranges between other satellites and the reference can be obtained as follows:

(8) $$\rho_{\text{ji},\text{rb}}=\lambda^{-1}\left(r_{\text{ji},\text{rb}}+I_{\text{ji},\text{rb}}+T_{\text{ji},\text{rb}}\right)+{ɛ}_{\text{ji},\text{rb}}$$

(9) $$\phi_{\text{ji},\text{rb}}=\lambda^{-1}\left(r_{\text{ji},\text{rb}}-I_{\text{ji},\text{rb}}+T_{\text{ji},\text{rb}}\right)+N_{\text{ji},\text{rb}}+\eta_{\text{ji},\text{rb}}$$

where the subscript “*ji*” represents the measurements of the *j*th satellites minus that of the *i*th (reference) satellite.

If there is a short baseline (e.g., 20 km) between the rover and the base, the ionospheric and delays of the two are assumed equal in this work, although they can be kept in the DD equations for further processing if one requires higher precision or needs to estimate ionospheric characteristics. Therefore, the DD *I* and *T* terms in Equations (8) and (9) are negligible and they can be rewritten as:

(10) $$\rho_{\text{ji},\text{rb}}=\lambda^{-1}r_{\text{ji},\text{rb}}+{ɛ}_{\text{ji},\text{rb}}$$

(11) $$\phi_{\text{ji},\text{rb}}=\lambda^{-1}r_{\text{ji},\text{rb}}+N_{\text{ji},\text{rb}}+\eta_{\text{ji},\text{rb}}$$

Equations (10) and (11) give the carrier phase and pseudorange measurement model under the short baseline condition. The baseline vector we need is buried in *r_ji_*_,rb_ terms. We expand *r_ji_*_,rb_ and reserve the first-order terms:

(12) $$r_{\text{ji},\text{rb}}=-\lambda^{-1}{\left({\text{a}}_{j,\text{r}}-{\text{a}}_{i,\text{r}}\right)}^{T}{\text{b}}_{\text{rb}}$$

where **a***_j_*_,r_ is the normalized line-of-sight (LOS) vector pointing from the rover to the *j*th satellite, and we assumed that the LOS vector of the rover equals that of the base under short baseline conditions, *i.e.*, **a***_j_*_,r_ = **a***_j_*_,b_. **b**_rb_ is the baseline vector pointing from the base to the rover.

With measurements from single or multiple epochs, the baseline vector **b**_rb_, the float DD ambiguity *N*_ji,rb_ and the ambiguity variance-covariance (VC) matrix can be obtained through a weighted least square method that is commonly found in literature, or a Kalman filter that is to be discussed in the following part. An integer searching method such as LAMBDA [12] can be employed to obtain the fixed integer ambiguities that are then used to correct **b**_rb_ to generate a high precision baseline solution.

## 3. Kalman Filtering for GPS/BDS Float Ambiguity Estimation

Kalman filtering can be used to estimate the baseline vector and the float ambiguities with a full utilization of the *a priori* knowledge of historical measurements. In this work, we select the three components of the baseline vector and the SD ambiguities as system states. It should be noted that the reason for selecting SD ambiguities rather than their DD counterparts is to avoid the handovers when the reference satellite changes, which is similar to the method in [13]. Of course, as an alternative, DD ambiguities can also be used as states to be estimated, and the reader can refer to Equations (36–39) in [14] to handle the changes of the reference satellite. If there are *m* satellites observed at a certain epoch, then the system states are expressed as follows:

(13) $$\text{X}={\left[x_{\text{rb}},y_{\text{rb}},z_{\text{rb}},N_{1,\text{rb}},N_{2,\text{rb}},\cdots ,N_{m,\text{rb}}\right]}^{T}$$

where *x*, *y* and *z* are the Cartesian components of the baseline vector.

One thing to note here is that the tracked satellite signal may disappear and new satellite may rise into view due to the receiver's dynamics, attitude change and environmental factors which may cause the number of ambiguities in the system states to vary. Measures including storing the rising and setting satellite indices should be taken to handle this situation and the reader can refer to [14].

If a receiver outputs both GPS and BDS measurements, jointly processing them with a Kalman filter may be beneficial for better positioning solutions. We should note that there is only one baseline at a certain epoch no matter which constellation is adopted. This fact produces correlations between the measurements of the two constellations that may not be fully exploited by handling GPS and BDS respectively. For this reason, we include all the SD measurements of both GPS and BDS as well as the baseline elements as the states of the Kalman filter as follows:

(14) $$\text{X}=\left[{\text{b}}_{\text{rb}}^{T},{\left({\text{N}}_{\text{float}}^{\text{GPS}}\right)}^{T},{\left({\text{N}}_{\text{float}}^{\text{BDS}}\right)}^{T}\right]={\left[x_{\text{rb}},y_{\text{rb}},z_{\text{rb}},N_{1,\text{rb}}^{\text{GPS}},N_{2,\text{rb}}^{\text{GPS}},\cdots ,N_{m,\text{rb}}^{\text{GPS}},N_{1,\text{rb}}^{\text{BDS}},N_{2,\text{rb}}^{\text{BDS}},\cdots ,N_{n,\text{rb}}^{\text{BDS}}\right]}^{T}$$

where **N**_float_ is the SD float ambiguity vector of GPS/BDS, the rover and the base have *m* common GPS satellites and *n* common BDS respectively, and **X** has *m* + *n* + 3 elements in total.

The baseline at *k* + 1 th epoch is modeled as the sum of the baseline components at *k*th epoch and a normally distributed random vector **W***_b,k_* as given in Equation (15):

(15) $${\text{b}}_{\text{rb},k+1}={\left[x_{\text{rb},k+1},y_{\text{rb},k+1},z_{\text{rb},k+1}\right]}^{T}={\text{b}}_{\text{rb},k}+{\text{W}}_{\text{b},k}$$

The ambiguities remain consistent between epochs unless cycle slip or/and satellite rising/setting happen. Therefore, the ambiguities at *k* + 1 th epoch equal that of the *k*th epoch:

(16) $$N_{j,\text{rb},k+1}^{*}=N_{j,\text{rb},k}^{*}$$

where the superscript “*” represents an arbitrary constellation.

The one-step state transition and measurement equations of the discrete Kalman filter can be written as:

(17) $${\text{X}}_{k+1}={\text{F}}_{k,k+1}{\text{X}}_{k}+{\text{W}}_{k}$$

(18) $${\text{Z}}_{k+1}={\left[{\text{ϕ}}_{\text{GPS}}^{T},{\text{ϕ}}_{\text{BDS}}^{T},{\text{ρ}}_{\text{GPS}}^{T},{\text{ρ}}_{\text{BDS}}^{T}\right]}^{T}=h\left({\text{X}}_{k+1}\right)+{\text{V}}_{k+1}$$

where **F***_k,k+_*_1_ is the one-step state transition matrix, **Z** is the DD carrier phase and pseudorange measurement vector, **W** and **V** are process and measurement noises respectively, the non-linear function *h*() as given in Equations (10) and (11) represents the relationship between the measurements and the states.

The one-step state transition matrix is an identity matrix as follows:

(19) $${\text{F}}_{k,k+1}={\text{I}}_{m+n+\text{3}}$$

By linearizing *h*(), the measurement matrix **H** can be obtained as given in Equation (20):

(20) $${\text{H}}_{k}=\left[\begin{array}{ccccc} {\text{H}}_{\text{bl},k}^{\text{GPS}} & {\text{D}}^{\text{GPS}} & & & \\ {\text{H}}_{\text{bl},k}^{\text{BDS}} & & {\text{D}}^{\text{BDS}} & & \\ {\text{H}}_{\text{bl},k}^{\text{GPS}} & & & \text{O} & \\ {\text{H}}_{\text{bl},k}^{\text{BDS}} & & & & \text{O} \end{array}\right]$$

where ${\text{H}}_{\text{bl},k}^{\text{GPS}}$ is the coefficient matrix of the baseline for GPS measurements, **O** represents a zero matrix and other matrices are similarly defined. Furthermore, based on Equation (12) and omit the subscript, we get Equation (21) where **a***_j_*_,r_ is the normalized LOS vector:

(21) $${\text{H}}^{\text{GPS}}=\left[\begin{array}{c} -\lambda_{\text{GPS}}^{-1}{\left({\text{a}}_{1,\text{r}}^{\text{GPS}}-{\text{a}}_{i,\text{r}}^{\text{GPS}}\right)}^{T}\\ \vdots \\ -\lambda_{\text{GPS}}^{-1}{\left({\text{a}}_{i-1,\text{r}}^{\text{GPS}}-{\text{a}}_{i,\text{r}}^{\text{GPS}}\right)}^{T}\\ -\lambda_{\text{GPS}}^{-1}{\left({\text{a}}_{i+1,\text{r}}^{\text{GPS}}-{\text{a}}_{i,\text{r}}^{\text{GPS}}\right)}^{T}\\ \vdots \\ -\lambda_{\text{GPS}}^{-1}{\left({\text{a}}_{m,\text{r}}^{\text{GPS}}-{\text{a}}_{i,\text{r}}^{\text{GPS}}\right)}^{T} \end{array}\right]$$

In Equation (20), **D** is the SD to DD transformation matrix for either GPS or BDS. It should be noted that the reference satellite cannot be a single satellite for both constellations because they have different signal carrier frequencies and their measurements cannot be mutually operated. As a consequence, we have to select two separated reference satellites from each constellation and double-difference the measurements within the single system itself. Take GPS as an example, if there are *m* common satellites and we choose the *i*th as the reference, then the **D**^GPS^ matrix can be written as Equation (22), and **D**^BDS^ can be derived similarly:

(22) $$\begin{array}{c} {\text{D}}^{\text{GPS}}={\left[\begin{array}{ccccccc} 1 & & & -1 & & & \\ & 1 & & -1 & & & \\ & & \cdots & \vdots & \cdots & & \\ & & & -1 & & 1 & \\ & & & -1 & & & 1 \end{array}\right]}_{\left(m-1\right)\times m}\\ i\text{th column} \end{array}$$

The Kalman filter equations can be written as follows:

(23) $${\text{P}}_{k+1|k}={\text{F}}_{k,k+1}{\text{P}}_{k}{\text{F}}_{k,k+1}^{T}+{\text{Q}}_{k}$$

(24) $${\text{K}}_{k+1}={\text{P}}_{k+1|k}{\text{H}}^{T}{\left(\text{H}{\text{P}}_{k+1|k}{\text{H}}^{T}+{\text{R}}_{k}\right)}^{-1}$$

(25) $${\text{P}}_{k+1}=\left(\text{I}-{\text{K}}_{k+1}\text{H}\right){\text{P}}_{k+1|k}$$

(26) $${\text{X}}_{k+1|k}={\text{F}}_{k,k+1}{\text{X}}_{k}$$

(27) $${\text{X}}_{k+1}={\text{X}}_{k+1|k}+{\text{K}}_{k+1}\left({\text{Z}}_{k+1}-h\left({\text{X}}_{k+1|k}\right)\right)$$

where **P***_k_* is the VC matrix of the states at *k*th update, **P***_k|k_*_+1_ is the one-step estimation of **P***_k_*, **K** is the Kalman gain matrix, **Q** is the process noise VC matrix and can be modeled as Equation (28), where the position related terms are set to infinite (usually a large number in practice) to reduce the dependence on receiver dynamic model, and the ambiguity related terms are set to zero based on its time-invariant feature:

(28) $$\text{Q}=\left[\begin{array}{ccc} \infty_{3\times 3} & & \\ & {\text{O}}_{m\times m} & \\ & & {\text{O}}_{n\times n} \end{array}\right]$$

**R** is the VC matrix of the DD measurements as given by Equation (29):

(29) $$\text{R}=\left[\begin{array}{cccc} {\text{D}}^{\text{GPS}}{\text{R}}_{\text{SD}}^{\phi ,\text{GPS}}{\left({\text{D}}^{\text{GPS}}\right)}^{T} & & & \\ & {\text{D}}^{\text{BDS}}{\text{R}}_{\text{SD}}^{\phi ,\text{BDS}}{\left({\text{D}}^{\text{BDS}}\right)}^{T} & & \\ & & {\text{D}}^{\text{GPS}}{\text{R}}_{\text{SD}}^{\rho ,\text{GPS}}{\left({\text{D}}^{\text{GPS}}\right)}^{T} & \\ & & & {\text{D}}^{\text{BDS}}{\text{R}}_{\text{SD}}^{\rho ,\text{BDS}}{\left({\text{D}}^{\text{BDS}}\right)}^{T} \end{array}\right]$$

The VC matrices of the SD GPS carrier phase and pseudorange measurements ( ${\text{R}}_{\text{SD}}^{\phi }$ and ${\text{R}}_{\text{SD}}^{\rho }$) are given in Equations (30) and (31) respectively, where *σ* is the variance of the original non-differenced base or rover measurements. The corresponding VC matrices of BDS can be obtained similarly:

(30) $${\text{R}}_{\text{SD}}^{\phi ,\text{GPS}}=\left[\begin{array}{ccc} {\left(\sigma_{\text{1},\text{b}}^{\phi ,\text{GPS}}\right)}^{2}+{\left(\sigma_{\text{1},\text{r}}^{\phi ,\text{GPS}}\right)}^{2} & & \\ & \ddots & \\ & & {\left(\sigma_{m,\text{b}}^{\phi ,\text{GPS}}\right)}^{2}+{\left(\sigma_{m,\text{r}}^{\phi ,\text{GPS}}\right)}^{2} \end{array}\right]$$

(31) $${\text{R}}_{\text{SD}}^{\rho ,\text{GPS}}=\left[\begin{array}{ccc} {\left(\sigma_{\text{1},\text{b}}^{\rho ,\text{GPS}}\right)}^{2}+{\left(\sigma_{\text{1},\text{r}}^{\rho ,\text{GPS}}\right)}^{2} & & \\ & \ddots & \\ & & {\left(\sigma_{m,\text{b}}^{\rho ,\text{GPS}}\right)}^{2}+{\left(\sigma_{m,\text{r}}^{\rho ,\text{GPS}}\right)}^{2} \end{array}\right]$$

Equation (27) contains the non-linear function *h*() of the baseline vector and LOS vector. The baseline vector and the distance from the receiver to a certain satellite can be estimated by single point positioning and calculating the satellite position with its ephemeris which is not discussed here; the reader can refer to any GPS textbooks such as [15].

## 4. GPS/BDS Combined RTK Implementation

**Figure 1.**
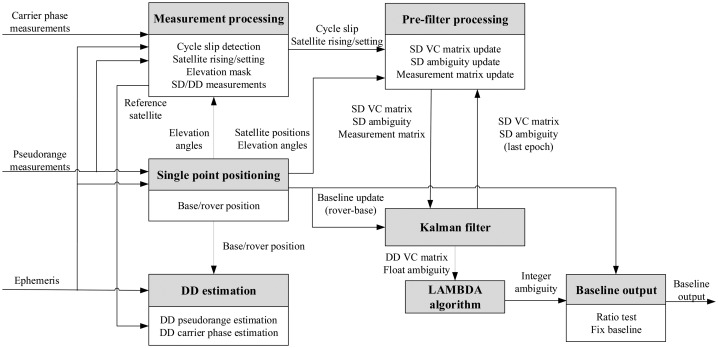
Flowchart of GPS/BDS combined RTK.

The implementation of the proposed GPS/BDS RTK model is divided into seven modules as Figure 1 shows, and are presented as follows.

(1) Single point positioning
  The pseudorange measurements from the rover and the base receivers as well as the broadcast ephemeris decoded by the rover receiver are used as the input to this module. An iterative least square (ILS) method as given in [16] (pp. 122–125) is employed to compute the position of the rover (denoted as **X**_r_) and the base (denoted as **X**_b_) receivers as well as the elevation angles of all visible satellites. The widely adopted Klobuchar model [17] and Saastamoinen model [18] are used to compensate the ionospheric and tropospheric delays respectively. When processing dual-constellation measurements, we use GPS to generate the single point positions in our implementation, although either constellation or their combination can be selected. It should be pointed out that the BDS time and GPS time have 14 s difference that has to be taken into account when calculating navigation solutions.
(2) DD estimation
  This module first generates the non-differenced carrier phase and pseudorange estimation using satellite positions from the ephemeris and the single point rover and base positions from the single point positioning module. The Saastamoinen troposphere model and the mapping function given in [19] are selected to reduce the DD residuals and produce more accurate estimations. Next we single-difference those estimations between the rover and the base, and then double-difference them between the reference satellite(s) and the other satellites. The estimated carrier phases and pseudoranges are denoted as **ρ**_DD_ and **ϕ**)_DD_ respectively.
(3) Measurement processing
  After the measurements have become available as inputs, some processing including detecting cycle slips (e.g., using a loss of lock indicator), handling satellite rising and setting, eliminating satellites below the elevation angle threshold, selecting reference satellite with the largest elevation angle and generating the DD measurements should be done.
(4) Pre-filter processing
  This module processes the float SD ambiguity **N**_float_ (containing both GPS and BDS ambiguities if handling dual-constellation) and the VC matrix **P***_k_* generated from the Kalman filter from the last epoch. If a cycle slip is detected for the *p*th satellite, the corresponding column and row of **P***_k_* will be zeroed, **P***_k_*'s corresponding diagonal element will be initialized and the *p*th element of **N**_float_ will be reset to the difference of the SD carrier phase and the SD pseudorange measurements, *i.e.*, Equation (7)−Equation (6). The initialization of the filter at the first epoch can be treated as an all-satellite cycle slip case, *i.e.*, all **P***_k_*'s diagonal elements and all **N**_float_'s elements should be initialized. If one new satellite rises, we should add a row and column to **P***_k_* and initialize the corresponding diagonal element, and **N**_float_ should be augmented with the corresponding term from Equation (7)−Equation (6) as well. If one satellite that existed at last epoch disappears at the current epoch, then **P***_k_* and **N**_float_ should be shrunk correspondingly. The measurement matrix **H** is generated in this module based on Equations (20) and (21). At each epoch, we also reset the baseline elements in the state vector to the difference of the single point positions of the rover and the base to avoid dependence on the dynamic features of the base or/and the rover, and reset the corresponding elements in **P***_k_* to a user-defined large number.
(5) Kalman filter
  Given the DD measurements as **Z** in Equation (27), and the updated **P***_k_*, **N**_float_ and **b**_rb_ from the pre-filter module, Equations (23)−(27) can be solved to produce the baseline, float SD ambiguities and the SD VC matrix for the current epoch. Here, *h*() in Equation (27) can be written as:
  (32) $$h\left({\text{X}}_{k+1|k}\right)={\left[{\text{ϕ}}_{\text{DD}}+\text{D}{\text{N}}_{\text{float}},{\text{ρ}}_{\text{DD}}\right]}^{T}$$
  where **D** is the transformation matrix from single to double-differenced measurements for either GPS or BDS as given by Equation (22), or to their combination as given by Equation (33):
  (33) $$\text{D}=\left[\begin{array}{cc} {\text{D}}^{\text{GPS}} & \\ & {\text{D}}^{\text{BDS}} \end{array}\right]$$
(6) LAMBDA algorithm
  The SD **N**_float_ and its VC matrix **P**_SD_ (the upper-left 4 ^∼^ 3 + m + n rows and 4 ^∼^ 3 + m + n column square matrix of **P***_k_*) from the Kalman filter can be transformed to their DD counterparts by **DN**_float_ and **DP**_SD_**D** that become the input to the LAMBDA algorithm [12]. The integer DD ambiguity **N**_int_ is obtained as the output of this module.
(7) Baseline output
  We use the popular ratio test [20] with a fixed threshold to decide whether to accept the integer ambiguity solution from LAMBDA. The final output of the baseline is given in Equation (34):
  (34) $${\text{X}}_{\text{bl}}={\text{X}}_{\text{r}}-{\text{X}}_{\text{b}}+{\left({\text{H}}_{\text{ϕ}}^{T}{\text{R}}_{\text{ϕ}}^{-1}{\text{H}}_{\text{ϕ}}\right)}^{-1}{\text{H}}_{\text{ϕ}}^{T}{\text{R}}_{\text{ϕ}}^{-1}\left({\text{ϕ}}_{\text{m},\text{DD}}-{\text{ϕ}}_{\text{DD}}-\text{N}\right)$$
  where ${\text{H}}_{\text{ϕ}}=\left[\begin{array}{c} {\text{H}}_{\text{bl}}^{\text{GPS}}\\ {\text{H}}_{\text{bl},k}^{\text{BDS}} \end{array}\right]$ is the coefficient matrix for baseline, **R**_φ_ is the DD carrier phase measurement VC matrix, **φ**_m,DD_ is the DD carrier phase measurement vector, and **φ**_DD_ is its estimation. If ratio test is passed, then **N** = **N**_int_ and the fixed baseline solution with higher precision is output, else **N** = **N**_float_ and the float baseline is output.

## 5. Experiment Setup and Result Discussion

**Figure 2.**
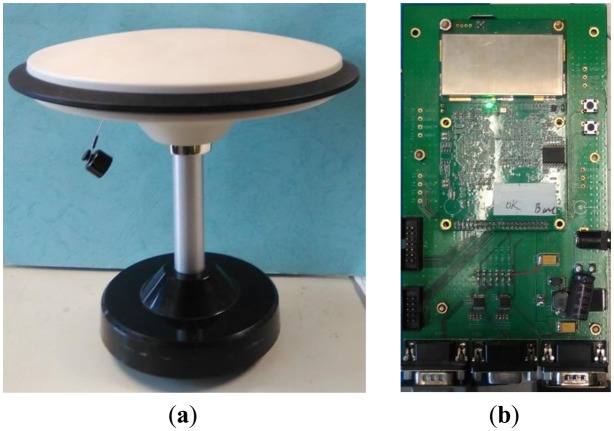
Experiment devices (**a**) antenna (**b**) receiver circuit board.

We use two self-developed GPS/BDS receivers as the base and the rover respectively. The receiver is developed based-on a DSP + FPGA architecture as shown in the right part of Figure 2. It can receive GPS L1/L2 and BDS B1/B2 signals via 96 parallel channels and can process L1 and B1 signals at this moment. We use a commercial survey antenna (left picture of Figure 2) and place it on the rooftop of Weiqing Building, Tsinghua University, Beijing, China.

**Figure 3.**
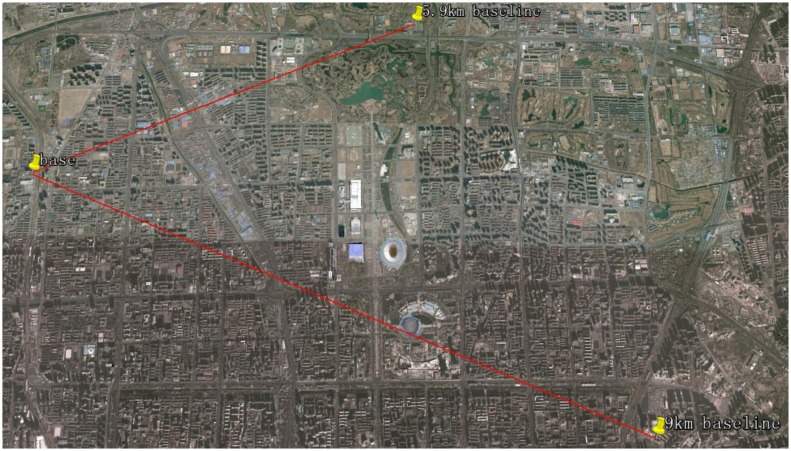
Base and rover locations (from Google Earth).

Three static experiments with a zero baseline, a 5.9 km baseline and a 9 km baseline for each are conducted. The zero baseline experiment is designed to verify the feasibility of the proposed model, and the other two short baseline tests are to evaluate its performance. In the zero baseline test, a radio frequency splitter was connected to the rooftop base antenna so as to split signals for the base and the rover receivers respectively. In the other two experiments, another antenna with the same performance as the base antenna was setup at an open-sky parking lot at the South gate of the North park of the Olympic Forest Park and the rooftop of the office building of China Academy of Civil Aviation Science and Technology respectively. The base and rover locations are drawn in Figure 3. The approximate locations calculated from pseudorange measurements for the base and the rovers in the tests as well as other experiment settings are listed in Table 1. The broadcast ephemeris and raw carrier phase and pseudorange measurements generated by the receivers are recorded during the tests. We implemented the proposed GPS/BDS combined RTK model on Matlab and post-processed the recorded measurements.

**Figure 4.**
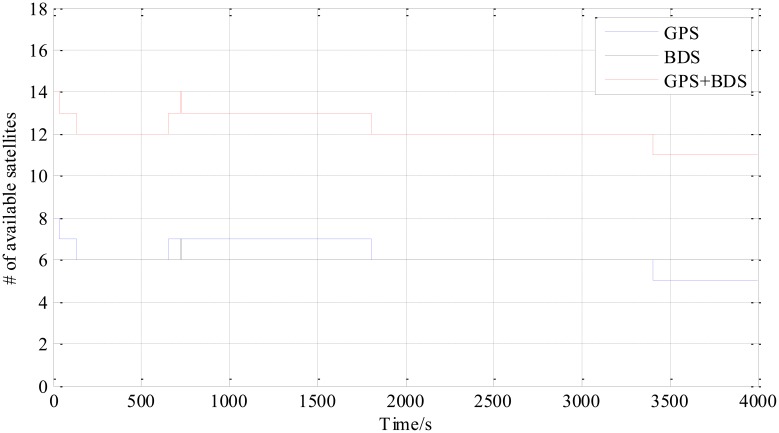
Number of available satellites *vs.* time.

**Figure 5.**
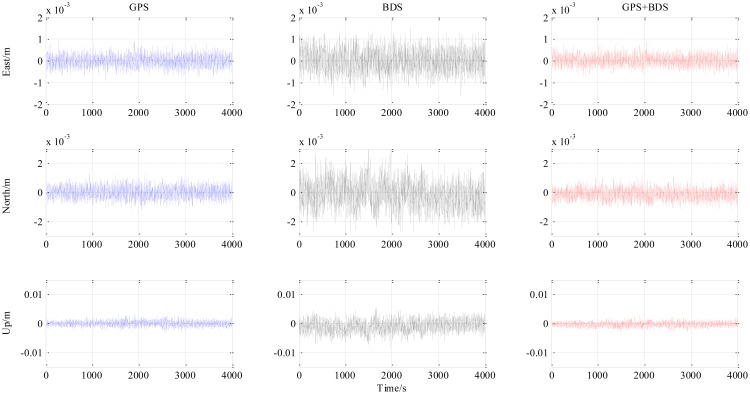
ENU baseline result of zero baseline experiment (all epochs).

Figure 4 is the number of available satellites during the testing period. Figure 5 shows the East, North and Up (ENU) positioning results of the zero baseline experiment. For comparison, the results of processing the single GPS and the single BDS with the same algorithm is included in the figure. Other statistics of the three methods are listed in Table 2. The result validates the feasibility of the proposed GPS/BDS RTK model and shows that the baseline precision of combined GPS and BDS is on the same level as that of handling either single constellation. The proportion of fixed solutions are 100% for all the three methods, *i.e.*, the ratios for all epochs reach above the threshold of 3. There is no difference in the first time to fix between the three approaches and they all enter fixed solution within one epoch. The reason for that should be the zero length of baseline.

**Figure 6.**
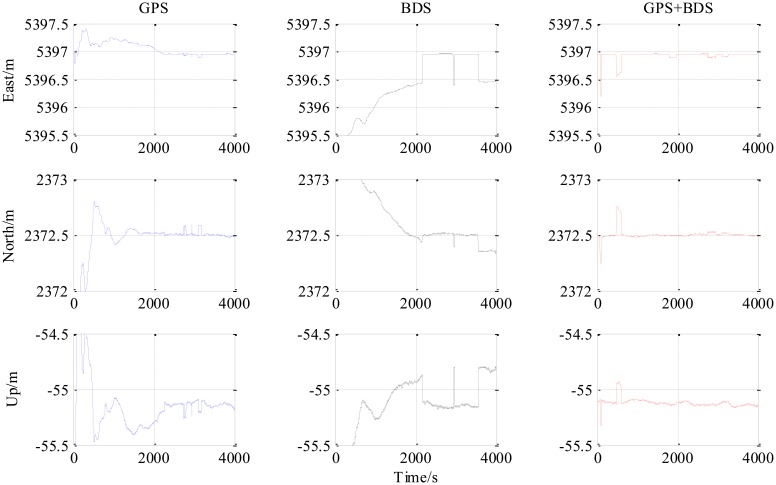
ENU baseline result of 5.9 km baseline experiment (all epochs).

**Figure 7.**
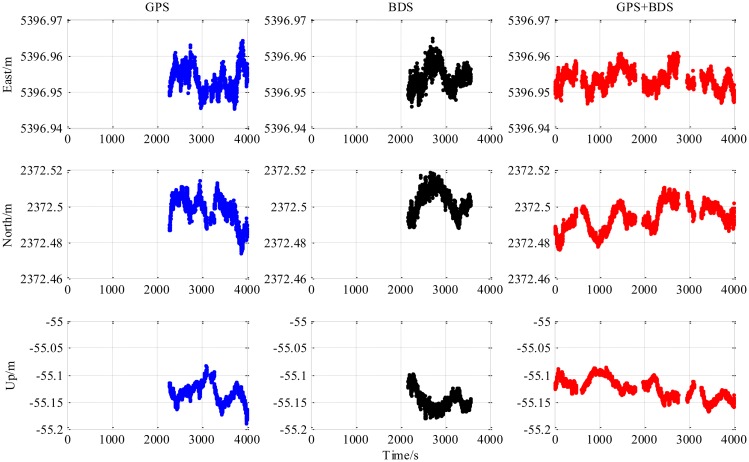
ENU baseline result of 5.9 km baseline experiment (fixed epochs, ratio > 3).

**Figure 8.**
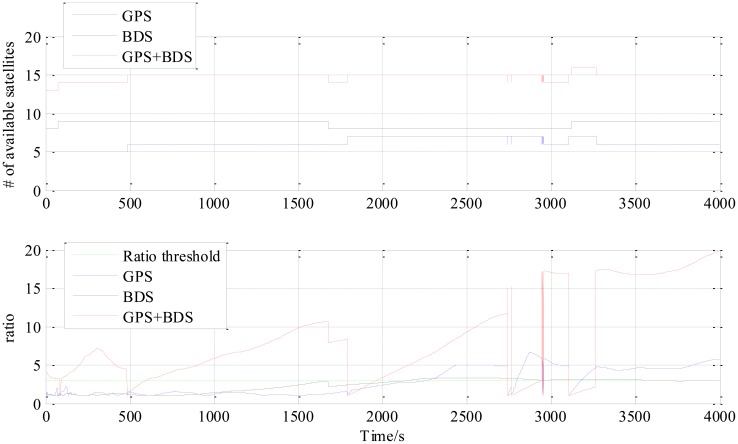
Number of available satellites and ratio *vs* time of 5.9 km baseline experiment.

The results from the 5.9 km short baseline test as shown in Figures 6, 7 and 8 and Table 3 illustrate some differences from the zero baseline experiment. First, the results demonstrate the effectiveness of the combined-constellation method. Besides that, the results of either single GPS or BDS take some time to enter a fixed solution while the combined method demonstrates a fast convergence as shown in the last row in Table 3. At the same time, the standard deviation as shown in the first three rows in Table 3 for all epochs of the combined method is much smaller than either single constellation method. Actually, for all epochs within the experiment, the overall standard deviation of GPS/BDS combined approach reaches 73.47 mm for East, 42.38 mm for North and 35.76 mm for Up which demonstrates an improvement over the other two methods. If we limit the scope to the fixed solutions, it is notable that all the three methods have similar precision level and the combined method does not show obvious improvement. Moreover, the proportion of fixed solution (ratio > 3) of the combined method (83.42%) is much higher than either single constellation method (39.98% and 34.08% respectively). This improvement shows that the GPS/BDS combination provides the higher availability of satellites and enhance the ability to enter fixed solution and therefore increase the availability of the RTK algorithm and its overall precision.

The “jumps” of ENU baseline solution in Figure 6 are transitions between the float and the fixed solutions for which we only use a hard decision by the ratio threshold to determine if the solution is fixed or not. The ratio values for the three methods shown in the lower part of Figure 8 sometimes fall below the threshold in a sharp manner, and it can be observed from the upper part of Figure 8 that the sharp falls of ratio below the threshold are coincident with new satellite rising. This indicates that the falls are caused by the new rising satellite because, in general, the new rising satellite has a low elevation angle which brings a high risk with relatively low signal-to-noise ratio or/and severe multipath effect. This fact makes the new coming measurements introduce inconsistency into the Kalman filter's VC matrix **P**_SD_, and causes the sharp falls of ratio.

**Figure 9.**
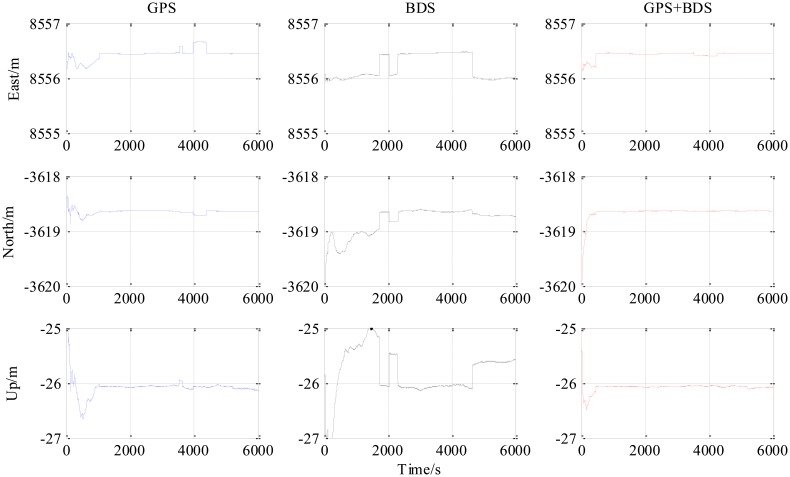
ENU baseline result of 9 km baseline experiment (all epochs).

**Figure 10.**
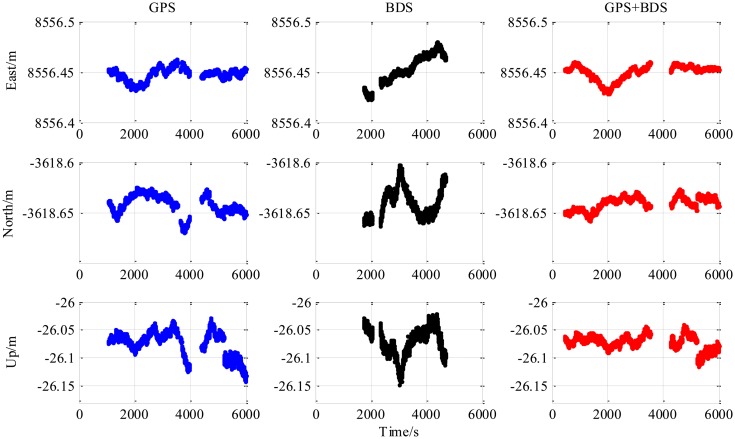
ENU baseline result of 9 km baseline experiment (fixed epochs, ratio > 3).

**Figure 11.**
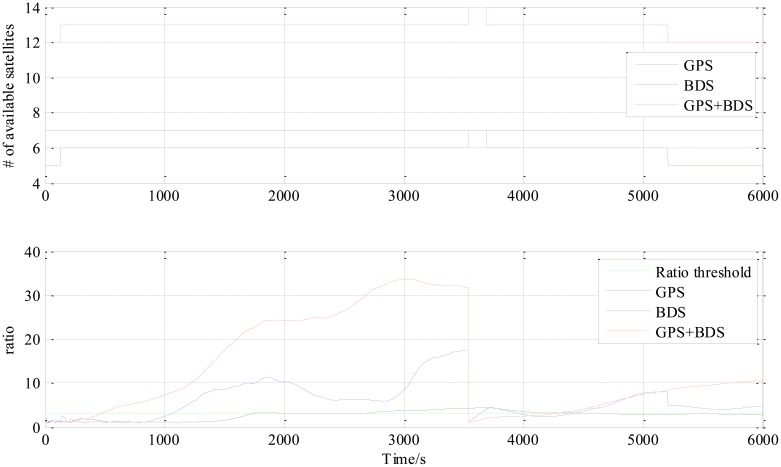
Number of available satellites and ratio *vs* time of 9 km baseline experiment.

The third experiment has a longer baseline (9 km) and a longer testing time (6000 epochs) than the 5.9 km baseline test. Baseline positioning results are shown in Figures 9, 10 and 11 (the variation of the number of satellites with time is shown in the upper part of Figure 11) and Table 4 which show some similarities to that of the 5.9 km baseline experiment. First, the fixed solution proportion of the combined method is higher than either using GPS or BDS. Besides that, a faster converging time to fixed solution is also obtained as shown in Table 4. However, compared with the 5.9 km baseline test, the results demonstrated some differences, e.g., the standard deviations of all the baseline components of GPS and BDS are slightly larger than that of the 5.9 km test which is possibly caused by the longer baseline. Another thing to notice is that the proportion of fixed solutions for either GPS or BDS in 9 km baseline test is higher than that of the 5.9 km baseline test. This is probably due to the smaller proportion of the converging time with a longer time span. However, there is still a gap between the fixed solution proportions of the combined method for the two short baseline tests to 100%, which indicates the limitation of the combined method—it cannot completely solve the problem of availability once and for all, some other measures should be investigated in future work.

## 6. Conclusions and Outlook

To benefit from more available navigation satellites due to the emergence of new GNSSs such as BDS, this work proposed a GPS/BDS single frequency combined high precision processing model. Kalman filtering is used to jointly process the carrier phase and pseudorange measurements from both constellations to estimate float ambiguities. The RTK algorithm for the dual constellations is discussed and the detailed work process and its implementation are presented by developing and integrating multiple functional modules. Readers can hopefully implement this algorithm without difficulty based on this contribution. The proposed method is validated via processing the field-collected single frequency carrier phase and pseudorange measurements of both GPS and BDS from a zero and two short baseline experiments. The experiment results show that the precision of the proposed method is comparable to the single constellation algorithm; the proportion of fixed solution based on the ratio test is improved and shorter time to first fix is obtained by using this combined method which extends the availability and reliability of the RTK algorithm.

Our future work will include adapting this method to multi-GNSS and multi-frequency applications with the consideration of its computational efficiency and testing its performance under dynamic environments. Other endeavors will lie in the measurement filtering and rejection strategy and optimization of fixed solution criterion that can help improve the solution precision, reliability and proportion of fixed solutions.

## Figures and Tables

**Table 1.** Experiment settings.

**Item**		**Zero Baseline**	**5.9 km Baseline**	**9 km Baseline**
Time (UTC)		11:48∼12:55	03:12∼04:19	12:41∼14:21
		13 May 2014	21 May 2014	31 July 2014
Frequency		GPS L1, BDS B1	GPS L1, BDS B1	GPS L1, BDS B1
Base	Latitude/°	40.0015	40.0015	40.0015
	Longitude/°	116.3302	116.3302	116.3302
	Height/m	98.9	98.9	98.9
Rover	Latitude/°	Same as Base	40.0228	39.9689
	Longitude/°	Same as Base	116.3934	116.4303
	Height/m	Same as Base	46.4	78.3
Data length/s		4000	4000	6000
Epoch interval/s		1	1	1
Elevation angle threshold/°		20	20	20
Ratio test threshold		3	3	3

**Table 2.** Statistics of zero baseline experiment.

**Item**	**GPS**	**BDS**	**GPS + BDS**
East standard deviation (fixed solution)/mm	0.30	0.48	0.26
North standard deviation (fixed solution)/mm	0.41	0.80	0.32
Up standard deviation (fixed solution)/mm	0.95	1.77	0.70
Proportion of fixed solution (ratio>3)	100%	100%	100%
First time to fix/s	1	1	1

**Table 3.** Statistics of 5.9 km baseline experiment.

**Item**	**GPS**	**BDS**	**GPS + BDS**
East standard deviation (fixed solution)/mm	3.60	3.20	2.67
North standard deviation (fixed solution)/mm	7.63	6.78	7.34
Up standard deviation (fixed solution)/mm	17.58	16.84	19.76
Proportion of fixed solution (ratio > 3)	39.98%	34.08%	83.42%
First time to fix/s	2277	2159	1

**Table 4.** Statistics of 9 km baseline experiment.

**Item**	**GPS**	**BDS**	**GPS + BDS**
East standard deviation (fixed solution)/mm	6.18	14.05	7.47
North standard deviation (fixed solution)/mm	9.80	14.12	6.40
Up standard deviation (fixed solution)/mm	22.52	25.17	12.55
Proportion of fixed solution (ratio > 3)	73.57%	44.25%	80.20%
First time to fix/s	1062	1734	467
